# Spatiotemporal variation in population size and relative abundance of Kalij pheasant (*Lophura leucomelanos*) in scrub forest

**DOI:** 10.1371/journal.pone.0312809

**Published:** 2024-12-30

**Authors:** Maria Saba, Sangam Khalil, Majid Hussain, Surrya Khanam, Ali Akhter, Rukhsana Khatoon, Muhammad Awais Khan, Kamal Ahmed Khan, Zartasha Gul, Ume Habiba

**Affiliations:** 1 Department of Forestry and Wildlife Management, The University of Haripur, Haripur, Pakistan; 2 Department of Forestry Range and Wildlife Management, The Islamia University Bahawalpur, Bahawalpur, Pakistan; 3 Department of Zoology, Women University, Sawabi, Pakistan; 4 Department of Wildlife Management, PMAS, Arid Agriculture University Rawalpindi, Rawalpindi, Pakistan; 5 Ministry of Climate Change, Wildlife Monitoring Office, Islamabad, Pakistan; 6 Department of Climate Change Forestry Environment and Wildlife, Khyber Pakhtunkhwa, Pakistan; 7 College of Life Sciences, Shandong Normal University, Jinan, China; 8 Department of Biology, The University of Haripur, Haripur, Pakistan; 9 Ministry of Climate Change, Islamabad Wildlife Management Board, Islamabad, Pakistan; Feroze Gandhi Degree College, INDIA

## Abstract

Most species of pheasants (Galliformes: Phasianidae) occur in Asia. In Pakistan, pheasants occur from 245–3050 m in altitude, and one of these, the Kalij pheasant (*Lophura leucomelanos*) is a large-bodied, brightly-colored habitat quality indicator species. The present study was designed to determine spatiotemporal variation, population size, and the relative abundance of Kalij pheasants in Haripur, Pakistan. The line transects and point count methods were used to collect direct (physical observations) and indirect signs (feathers and calls) to avoid double counting. Twenty field surveys were conducted along 16 transects with an area of 0.811 km^2^ in 4 study sites to quantify habitat utilization of Kalij pheasant. Kalij pheasant activities were observed at the start of dawn (0600 hours to 0730 hours) and ended by 1630 hours to 1800 hours dusk period with only a few sightings during the middle of the day. Study sites were selected based on confirmation of presence signs. Twenty-seven individuals with direct (16) and indirect (feather = 5, calls = 6) signs were encountered while walking along line transects. Twelve signs were counted (sightings = 3, Calls = 9) within the radius of 11–50 m in the point count method. Results showed that the Kalij pheasant occupied scrub forest with dense vegetation between 500-1100m away. The mean encounter rate of the Kalij pheasant was 0.82/km, which was higher from July to September. The sex ratio (1.17) is biased toward males in the overall population of Kalij pheasants. The estimated mean density of the Kalij pheasant in Haripur was 6.97 animals/km^2^, and the total abundance was 142 individuals. New potential sites may be identified and declared as protected areas, and awareness and support of the local community are recommended for the protection and management of the Kalij pheasant.

## Introduction

The order Galliformes (game birds) is an important bird group worldwide. Within this group, pheasants (family Phasianidae) are habitat quality indicators as they are heavily dependent on understory and surface vegetation and sensitive to environmental quality fluctuations [1]. Due to their plumage and meat, pheasants are, therefore, of significant importance and economic benefit to human communities [2]. So far, 51 species belonging to 16 genera have been recognized globally. Interestingly, 50 of them are Asian in origin [3].

The Kalij pheasant (*Lophura melanoleucos*) has a wide distribution in Asia, from the Himalayas of Afghanistan to Pakistan in the west, continuously through north India, Nepal, northeast Tibet, and China [4]. This bird is thought to have a significant global population and is presently considered a species of low conservation concern [5]. The Kalij Pheasant is difficult to observe, as it lives in dense undergrowth at elevations of 2100–3,300m in temperate broadleaf, conifer, and subalpine oak woods [6]. Within Pakistan, Kalij Pheasants are found in Khanpur, Makhniyal, Kashmir, Swat and Kohistan. It spreads eastward to Kashmir via the Siran Valleys and Kaghan valleys [7]. The Kalij pheasant is sexually dimorphic as females have a shorter crest with no lateral erectile feather tufts, while males have a longer crest with lateral erectile feather tufts. The head of both sexes is completely feathered [8]. The breeding season is from February to October but mainly from April to May. The Kalij pheasant (*Lophura leucomelanos*) is said to be bred from March to May [9].

All pheasants face many threats related to population explosion intrusion (human disturbance, habitat loss, urbanization, poaching, and diseases) [10]. The Kalij pheasant population is declining due to overhunting for its plumage and meat and egg damage during the breeding season [11].

The study area, i.e., Haripur, is vast and less explored in terms of spatiotemporal variation and relative abundance of Kalij Pheasant. The first baseline study of Kalij pheasants on status habitat use variation and abundance has been designed as no previous research has been conducted to date. The wildlife department will use this information for future planning and conservation of this species in the study area.

## Materials and methods

### Study area

**Fig 1:**
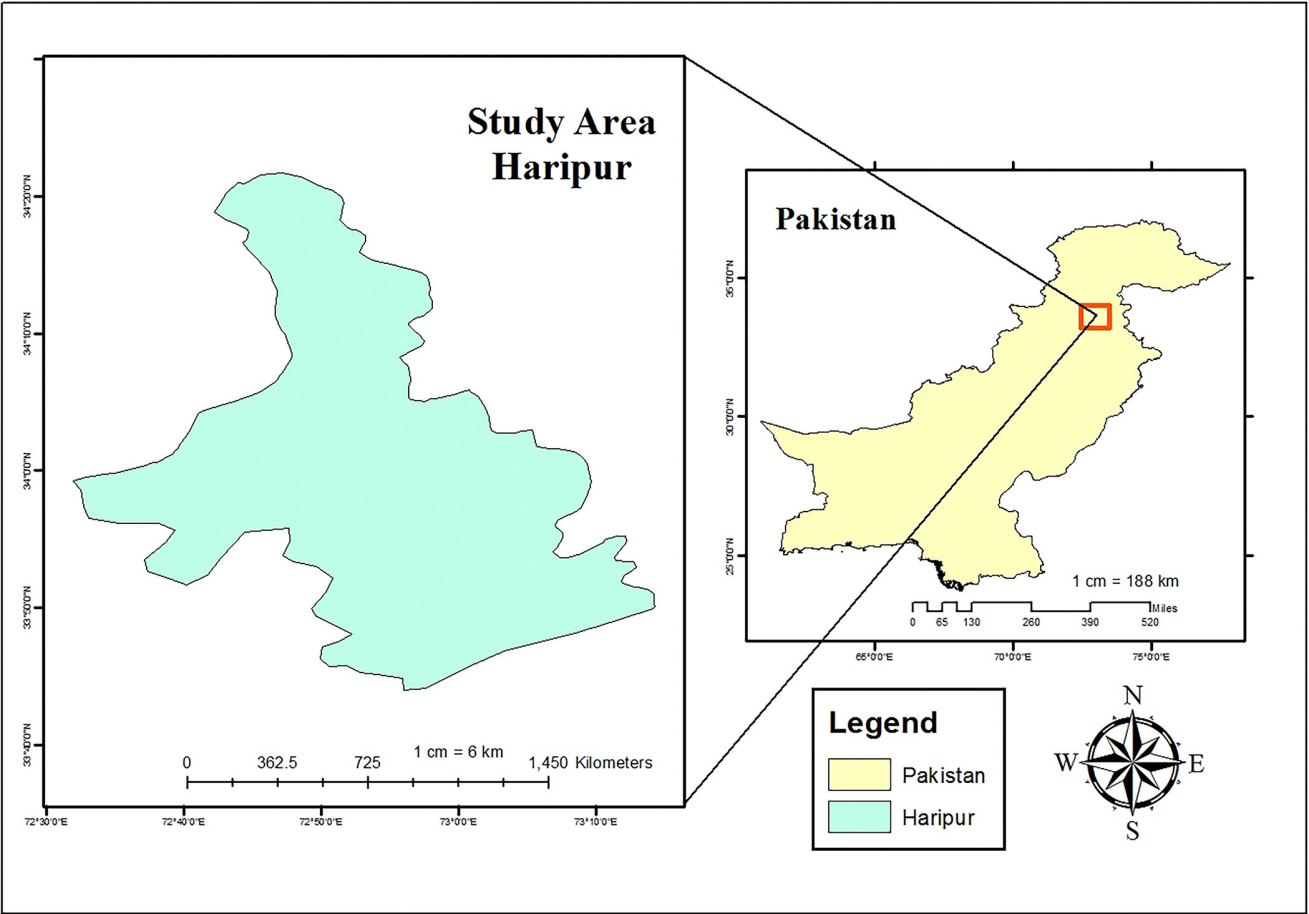
Map of the study area.

The present study was conducted in the district of Haripur, which lies at an altitude of around 610 meters above sea level (Fig 1).

The town of Haripur was founded in 1822 by Hari Singh Nalwa, the Commander-in-Chief of Ranjit Singh’s army. It has an area of 1,725 km^2^ and is the 45th largest city in Pakistan by population, according to the 2017 census [12].

Currently, Haripur District is divided into three tehsils, Haripur Tehsil, Khanpur Tehsil, and Ghazi Tehsil, further subdivided into 45 Union Councils, of which 15 are urban Union Councils. The area is rich in natural resources and contains two reservoirs, the Tarbela Dam and Khanpur Dam. Geographically, it is the gateway to Hazara, the Hazara Division [13].

The average minimum and maximum temperatures of Haripur are 3° C and 41° C, respectively. January is the coldest month, and June is the hottest month. An average of 19–70% humidity can be recorded throughout the year. The annual average precipitation at Haripur is 74–1260 mm [14]. The study area consists of prominent plant species from scrub forests. The main tree species are Gond (*Eucalyptus mannifera*), Kachnar (*Bauhinia variegata*), Shisham (*Dalbergia sissoo*), Phulai (*Acacia modesta*), Olive (*Olea ferruginea*), and Paper Mulberry (*Broussonetia papyrifera*). Characteristic bird and mammal species in District Haripur include the Kashmir Flying squirrel (*Eoglaucomys fimbriatus*), Indian Crested Porcupine (*Hystrix indica*), Rhesus Monkey (*Macaca mulatta*), Yellow-billed blue magpie *(Urocissa flavirostris)*, Koklass pheasant *(Pucrasia Macrolopha)*, Kalij pheasant (*L*. *leucomelanos)*, Tree pipit (*Anthus trivialis*), Red-vented bulbul (*Pycnonotus cafer*), Black kite (*Milvus migrans*), Grey francolin (*Ortygornis pondicerianus*), House sparrow (*Passer domesticus*), etc. In minimal numbers, Chukar *(Alectoris chukar)* and Common peafowl (*Pavo cristatus*) are also reported [15].

### Spatiotemporal variation

The study was designed to be conducted from July to December 2021 to accomplish the objectives proposed. The study area was surveyed thoroughly to check for Spatial-temporal variation in the population size of Kalij pheasant.

In the reconnaissance survey, we generated an idea about the distribution and potential sites of the Kalij pheasant based on confirmation of direct (sighting of the Kalij pheasant) and indirect signs (footprint, feather, and call) in tehsil Khanpur. The field guide was used to identify species coordinates, and the elevation of Kalij pheasant occurrence sites was noted using GPS.

Although attempts were made to gather information on spatial distribution and occurrence patterns for Kalij pheasant, available data based on direct and indirect sign data collected during this study and other secondary sources [16] and interviews with local people indicated that Kalij pheasant had sparse distribution in Haripur. Intensive studies were conducted in four potential study sites, i.e., (1) Makhniyal, (2) Karwali, (3) Khanpur (4) Soha. Each study site has different characteristics (Table 1).

**Table 1:** Characteristics of each study site selected during the survey in the study area.

Site number	Site name	Elevation (m)	Transect Length (km)	Slope %	Level of Disturbance	Observations
1	Makhniyal	960–1100	8.1	75–80	Low	12
2	Karwali	875–950	7.7	50–60	Low	4
3	Khanpur	650–800	6.7	50–60	Medium	9
4	Soha	450- 640m	7.9	30–40	High	2

### Line transect method

We surveyed Kalij pheasant using line transects [17]. This method involved walking on existing trials and counting the birds and signs encountered on both trail sides. The height above ground from the line transect to the collected sign was measured in meters [18].

A total of 16 trails ranging from 2–5 km with different widths (10-30m) depending upon the condition of potential sites were walked sequentially twice a month, and the encounter rate was estimated. Data on species encounter number, sex, and sighting distance was recorded for further analysis. Encounter rate (ER) is expressed as the number of individuals seen per unit effort [19].

ER=n/L,

Whereas n = number of sightings or birds detected, L = distance covered.

The distance sampling technique is widely used for determining the abundance and densities of various wildlife species [20]. Distance software version 7.3 was used to analyze and estimate the relative abundance, population density, encounter rate, and detection probability of the Kalij pheasant population using line-transect data [21].

### Point count method

Point counts with various radii were laid out randomly in the study area.” E.g., “We also measured the relative abundance of Kalij pheasants by hearing calls or sighting birds at variable-radius point counts laid out randomly in the study area [22]. Point counts with various radii were laid out randomly in the study area. The radius was varied depending on the habitat types. The number of individuals of each species was detected within a 20-35m radius. Bird distance was recorded from the categories (0-25m, 25-50m, and >50m). Birds that originally were detected outside the 20-25m radius boundary but letter moved within 25m were recorded as occurring within the fixed radius circle.

### Spatial distribution

Transects were placed in different habitats, such as disturbed areas, undisturbed areas, open canopy areas, and cultivated, mixed forests, to examine the habitat use of the Kalij pheasant. Twenty field surveys were conducted with three visits to each of the four study sites during the study period from July to December 2021. A 14 km^2^ area of Kalij pheasant habitat was identified to be occupied. A total of 16 transects with an area of 0.811 km^2^ were laid during the study period.

## Results and discussion

### Spatial distribution

Direct observation was used extensively to investigate habitat utilization, social structure, and occurrence patterns. By and large, 27 individuals were encountered, which included direct (16) and indirect (feather = 5, Calls = 6) signs while walking along line transects (Table 2**)**. Selvan et al. [17] also reported findings similar to those of the present study, which found that 28 sightings of Kalij pheasants were made in India.

**Table 2:** Record of observations in each study site from July to December 2021.

S. No.	Site name	Sightings	Calls	Feathers	Area of transect (km^2^)
1	Makhniyal	7	2	3	0.162
2	Karwali	3	1	0	0.185
3	Khanpur	4	3	2	0.24
4	Soha	2	0	0	0.224
Total		16	6	5	0.811

While walking along transects and selecting point counts, it was noted that the Kalij pheasant was presently associated with a different set of habitat factors at the macro and microhabitat levels. It was detected directly in all four study sites surveyed during the study period, but no indirect sign was noted in one study site named Soha. Makhniyal was the most suitable habitat for the Kalij pheasant, followed by Khanpur based on the record of sign data (Table 2).

Apart from monitoring trails and transects, the point count method and call counts [16] were carried out at each site to estimate Kalij pheasant abundance. Suitable vantage points (hilltops and facing valleys) were selected, seven at each site (total of 28) to record the sightings and call individuals from different directions.

The vantage points were attended for half an hour at dawn and dusk. A count within 50m of the designated location was done for safety reasons. It was made sure all points were at least 300m apart to avoid doubling and biased counting. A total of 12 signs were counted, which included (sightings = 3 and calls = 9) within a radius of 11–50 m (Table 3). In contrast, the maximum number of sign data was found between 21–40 m radii. No feather within the radius of the vantage point was noticed.

**Table 3:** Records of observations of Kalij pheasant by point count method in the study area.

Radial Distance (m)	No. of observations	Sightings	Calls	Feathers
0–10	0	0	0	0
11–20	1	0	1	0
21–30	3	1	2	0
31–40	7	2	5	0
41–50	1	0	1	0

### Spatiotemporal variation

#### Relationship with vegetation composition

**Fig 2:**
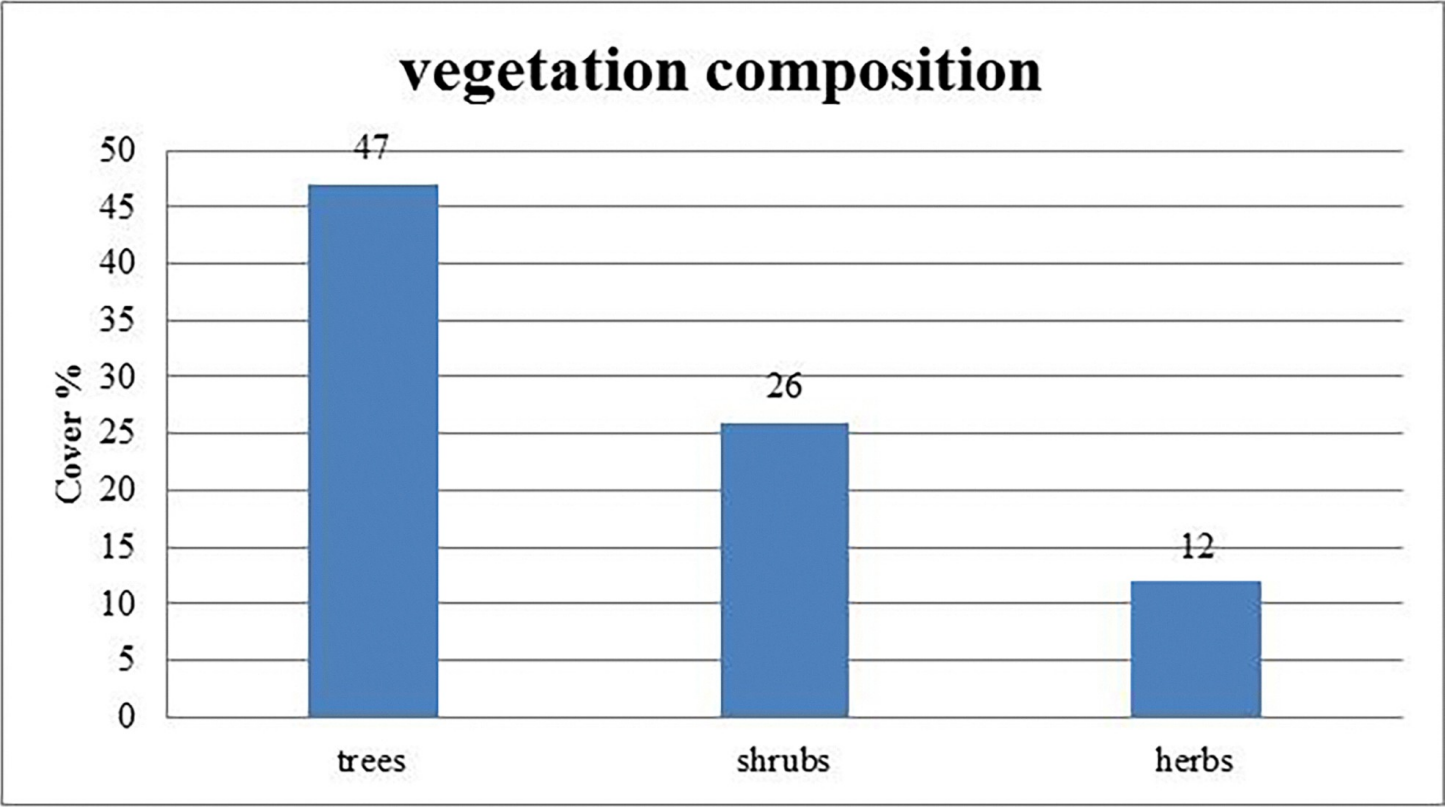
Vegetation composition of Kalij pheasant habitat in the study area.

It was observed that the Kalij pheasant was not continuously distributed in every area of Haripur but mainly occupied the scrub forest adjacent to Margalla Hills National Park with water supplies and suitable feeding areas. Combined with the dense vegetation, the number of trees and shrub cover had made a positive association with Kalij pheasant detections (Fig 2).

Similar results were reported by Selvan et al. [17], who stated that Kalij pheasant detection was high in low canopy cover and high tree density areas. Sathyakumar et al. [18] also documented that Kalij pheasant mostly preferred moderate grass cover, tree cover, and shrub cover in the Himalayas, which is similar to this study’s finding.

#### Relationship with elevation

**Fig 3:**
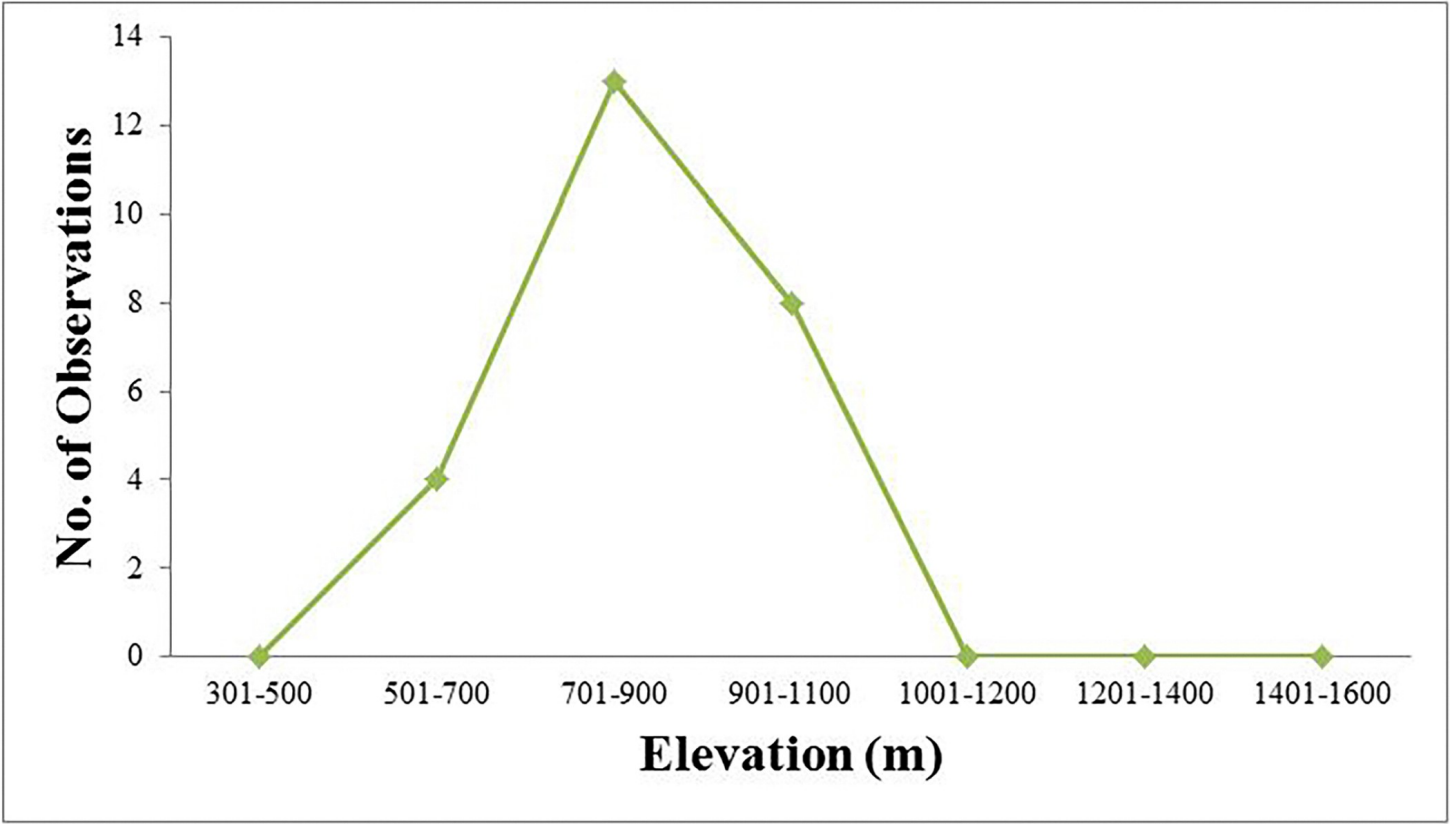
Kalij Pheasant occupies the elevation range in the study area.

Of the 27 sightings recorded for Kalij pheasant, a suitable forest habitat was a medium-altitude broad-leaved forest between 500–1100 m away from human habitation (Fig 3). Similar results were reported by Lewin and Lewin [21], who revealed that 95% of Kalij’s sightings were between 450 and 2150 m. Gaston et al. [16] results contradicted the findings of this study, where he observed Kalij pheasant mostly between 1600 and 2500 m.

#### Relationship with the degree of disturbance

The disturbance was negatively associated with Kalij pheasant detections (Table 1). Bhattacharya et al. [23] also reported similar results, stating that the presence of humans and livestock has a negative impact on the Kalij pheasant. The negative association of Kalij pheasant with human disturbance corresponded to Hussain et al. [24] findings that contradicted Gaston et al. [25] observations.

### Activity pattern

The date, time, species, and the activity first sighted were recorded on the datasheet. The total number of the group and numbers in different sex and age classes were also noted. There were 16 transect monitoring points and 28 points involving 128 hours of search efforts during the entire study period, which resulted in the sightings of 39 individuals altogether using the two methods.

**Fig 4:**
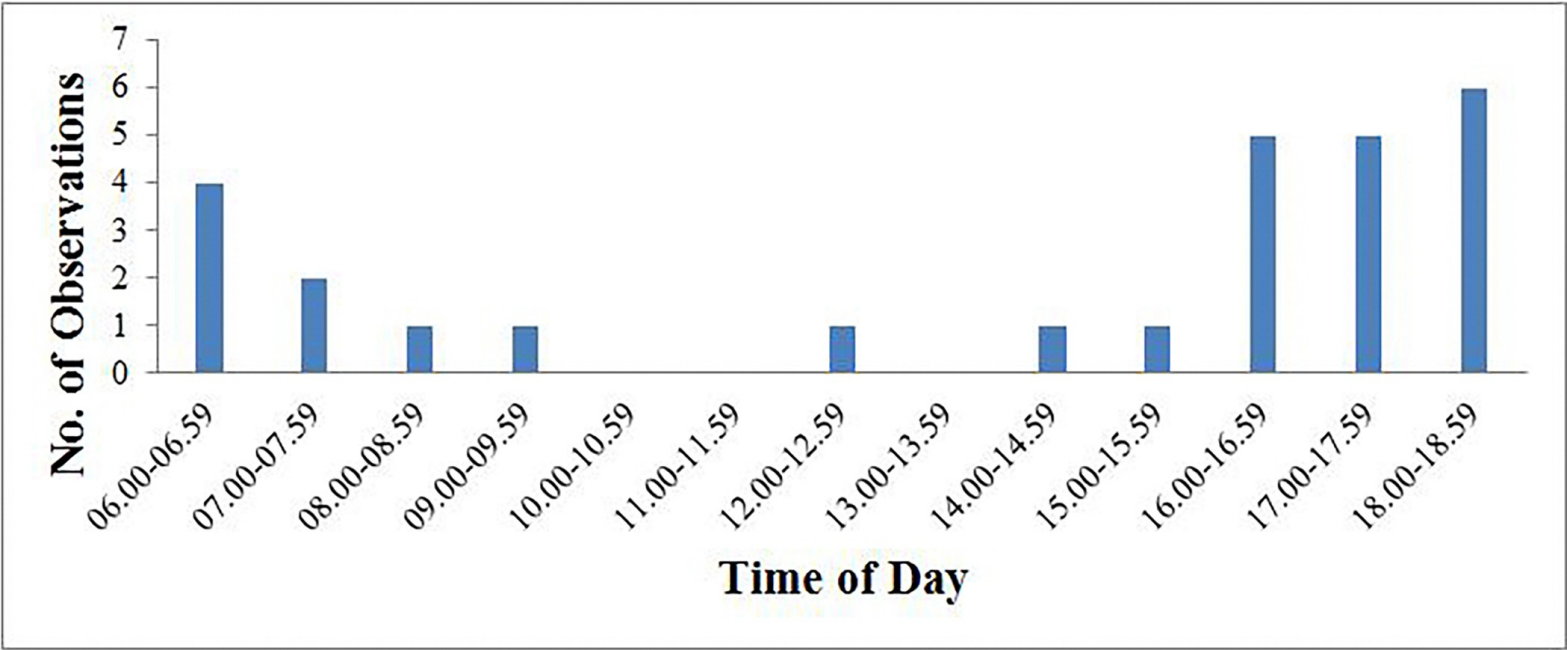
Sighting frequencies of Kalij pheasant by time (Pakistan standard time; PST).

Kalij pheasant activities were observed at the start of dawn (0600 hours to 0730 hours) and ended by 1630 hours to 1800 hours dusk period with only a few sightings during the middle of the day (Fig 4).

Results were contradictory to the findings of Selvan et al. [17], where activities were observed before dawn from 0400 hours to 0430 hours. Activities of the Kalij pheasant were not noted before dawn as it was complicated to reach the field early this morning due to culture and topographical features.

More than 70% of temporal overlaps occur of Kalij pheasant between 0600–0800 hours and in the evening between 1600–1800 hours (Fig 4). The sighting frequencies of the Kalij pheasant were highest from July to October when it was dry and hot. Kalij Pheasants had early morning and late afternoon peaks of foraging with lower levels at mid-day. Lewin and Lewin [21] reported the exact timings for activities of the Kalij pheasant and documented that this is a typical activity pattern of most game birds. Kalij pheasant activity was very low during the morning hours compared to the evening. After 1000 hours, movements slowed down, though few calls of the Kalij pheasant were heard during the daytime.

### Population size

#### Encounter rate

Kalij pheasant was encountered differently in the time of year, with a mean counter rate of 0.82/km. As July and August were thought to be the breeding season of Kalij pheasant, activity level was considered different. The encounter rate was significant from July to September (Table 4).

**Table 4:** Variation of encounter rate of Kalij pheasant in different months.

Months	Length (km)	Number of observations	Encounter Rate/Km
July	5	8	1.6
August	7	9	1.29
September	4	7	1.75
October	3	1	0.33
November	6	0	0.00
December	5	1	0.2
Mean Encounter rate			0.82

### Sex and age ratio of Kalij pheasant

Data on the distribution of Kalij pheasant in the study area indicated a higher number of males in most seasons and study sites. The sex ratio (1.17) is biased toward males in the overall population of Kalij pheasants (Table 5). Males in the population were easily detectable by calls and direct observation (colorful plumage). Selvan et al. [17] determined Sex ratio of Kalij pheasant was 1.2 (1:1.2) females per male, which matches the finding of this study.

**Table 5:** Sex and age composition of Kalij pheasant in the study area.

S. No	Site name	Male	Female	Chick	Male/Female Ratio
1	Makhniyal	4	3	0	1.33
2	Karwali	2	1	0	2
3	Khanpur	1	1	2	1
4	Soha	0	1	1	0
Total		7	6	3	1.17

The encounter of chicks with females showed that the study area’s population is breeding and could increase in number if protected. Direct sightings calculated the sex ratio in transects, opportunistic sightings, and photographs obtained from the camera traps. Females were higher than males for Kalij pheasant, similar to Javed and Rahmani’s [26] findings.

### Group size

**Fig 5:**
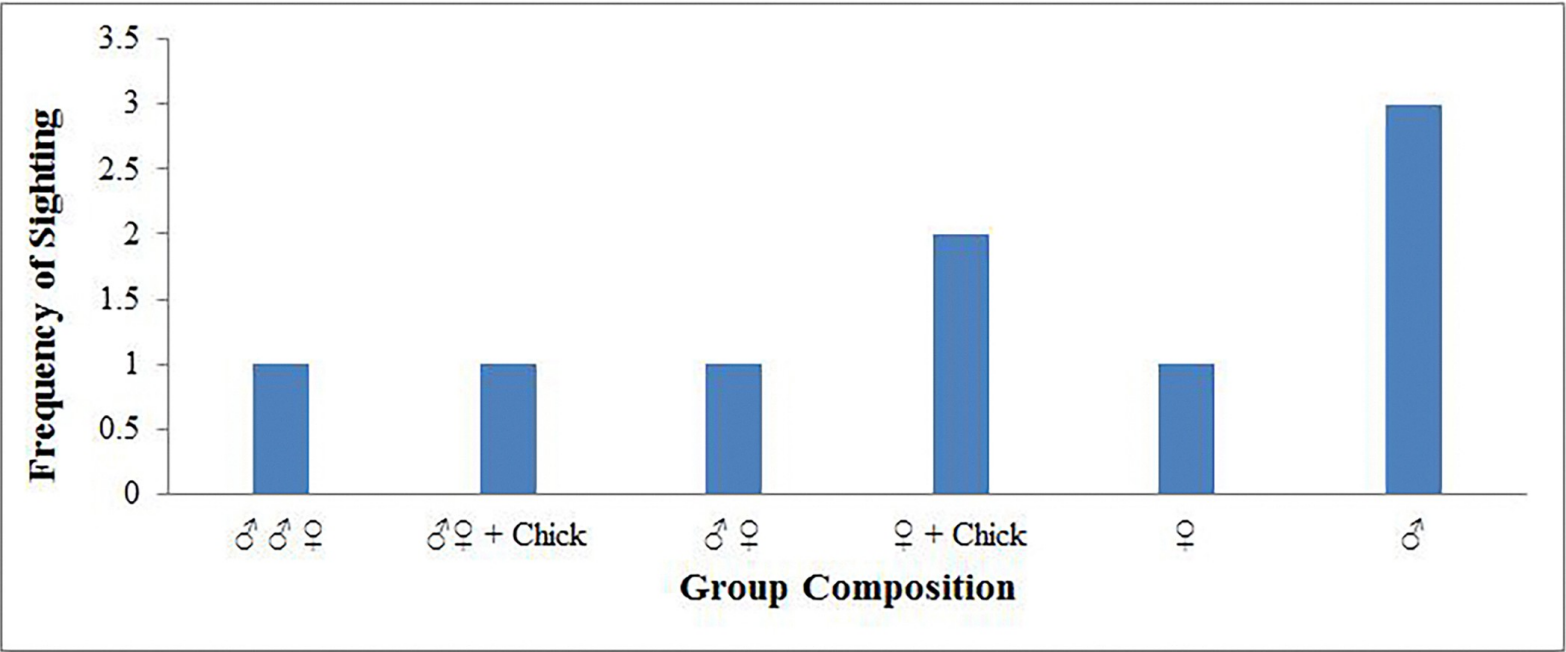
The group size composition of Kalij pheasant is in the study area.

The group consisting of three individuals was the maximum group size observed only two times for this species throughout the study period. Other group size characteristics of Kalij pheasant have been given (Fig 5).

Sightings of different groups also indicated that males and females occupied the same elevation and time-activity pattern. No elevation shift was observed in the Kalij pheasant in the study area. One sighting of a male with two female birds during the breeding season indicated a possible ambiguity of the general observations that they are strictly monogamous.

### Relative abundance

#### Selection of models

They were based upon a combination of Akaike’s Low Information Criterion (AIC) and detection function. The first three models were considered to estimate abundance and density. The coefficient of Variance in estimated Kalij pheasant density was calculated, and log-normal 95% confidence intervals were calculated for each model’s abundance and density estimates as described in Buckland and Elston [27]. Kalij pheasant abundance was obtained by multiplying the final density estimate by the habitat area (Table 6).

**Table 6:** Estimation of density and relative abundance of Kalij pheasant in the study area from July to December 2021.

S. No.	Model	AIC value		Estimate	% CV	Df	95% Confidence Interval Upper Lower
1	CDS Negative Exponential-Cosine	117.55	D	6. 8	62.17	16.23	2.02 22.74
			N	138	62.17	16.23	41 464
2	CDS Hazard rate-Cosine	119.29	D	7.3	21.93	23.60	4.65 11.40
			N	149	21.93	23.60	95 233
3	MCDS Halfnormal-Hermite polynomial	117.55	D	6.8	37.91	18.45	3.14 14.62
			N	138	37.91	18.45	64 298
4	MCDS Hazard rate-Cosine	119.29	D	7.3	21.93	23.60	4.65 11.40
			N	149	21.93	23.60	95 233
5	MCDS Hazard rate- Polynomial	119.29	D	7.3	21.93	23.60	4.65 11.40
			N	149	21.93	23.60	95 233

CDS = Conventional Distance Sampling, MCDS = Multiple Covariate Distance Sampling

AIC = Akaike’s Low Information Criterion CV = Coefficient of Variance, df = Degree of Freedom.

Initially, the data were analyzed because no prior information on Kalij pheasant densities was available for the study area. Models of abundance and density were chosen based on how well the probability density functions fit the line transect observations.

### Population density and abundance

According to the Distance software, the estimated mean density of the Kalij pheasant in Haripur was 6.97 animals /km^2.^ The total abundance was 142 individuals of the Kalij pheasant, with a maximum encounter rate and probability of detection. Selvan et al. [17] also reported findings similar to those of the present study, which estimated the density of Kalij pheasant to be 6.7/km^2^.

## Conclusions

Micro-habitat factors associated with Kalij pheasant are very limited in the study area. The Kalij pheasant occupied few patches of available habitat. Although mean density and abundance were found to be satisfactory in the study, they were also declared as least concern on the IUCN Red List of threatened species. The population and habitat of Kalij pheasant are decreasing due to illegal hunting for meat, deforestation, road building, grazing operations, noise pollution, and encroachment of land for agriculture. Hence, prompt attention is needed to allow appropriate conservation efforts.

The Khyber Pakhtunkhwa government is now paying more attention to wildlife protection. It has declared the habitat of the Kalij pheasant a Special Heritage Site. It needs to upgrade laws and regulations to protect animals and their habitats so that anyone who hunts, sells, or sells illegally will be poorly prosecuted.

Community-based guides also enhance community involvement in the protection of Kalij pheasant. KP Department of Wildlife is now participating in a pilot project with the communities that are monitoring resources. We believe that the results of this study also highlighted as much as possible the consideration of the local community’s participation as a standard tool for monitoring demographic trends in the medium and long term.

### Recommendation

Based on the findings of this research study, the following recommendations are made:

1. Habitat has a significant impact on Kalij pheasant, so their habitat (food, water, shelter) must be conserved. Deforestation also disturbs wildlife because of the forest area, particularly the habitat of the Kalij pheasant. So, there is tremendous pressure on our forest that people from the regions fulfill their domestic requirements from this forest. They have a very big role in the destruction and disturbance of Kalij pheasant. The Govt. should provide the best alternative to the people of these regions to shed the pressure on these forests to ensure biodiversity.

2. Awareness should be created among the people through school conservation clubs. Publicity boards carrying conservational notes, slogans, and information related to the Kalij pheasant may be prepared and erected.

3. Community organizations play a crucial role in conserving and sustainable development of natural resources. The initiative will support meetings and workshops at the community level to enlist the support of the local community for Kalij pheasant management.

4. New potential sites may be identified, and they may be declared protected areas. It will bring more areas under protection and conserve Kalij pheasant in the respective places on a sustainable basis.

5. A system of rotational grazing should be adopted to ensure proper management of rangelands. Routes may be identified and fixed for the movement of livestock in the study area.
